# Nanostructures for Light Trapping in Thin Film Solar Cells

**DOI:** 10.3390/mi10090619

**Published:** 2019-09-17

**Authors:** Amalraj Peter Amalathas, Maan M Alkaisi

**Affiliations:** 1 Centre for Advanced Photovoltaics, Faculty of Electrical Engineering, Czech Technical University in Prague, Technická 2, 16627 Prague, Czech Republic; 2 Department of Electrical and Computer Engineering, University of Canterbury, Christchurch 8140, New Zealand; maan.alkaisi@canterbury.ac.nz; 3 MacDiarmid Institute of Advanced Materials and Nanotechnology, Wellington 6140, New Zealand

**Keywords:** light trapping, solar cells, thin films, photonic nanostructures, plasmonic nanostructures

## Abstract

Thin film solar cells are one of the important candidates utilized to reduce the cost of photovoltaic production by minimizing the usage of active materials. However, low light absorption due to low absorption coefficient and/or insufficient active layer thickness can limit the performance of thin film solar cells. Increasing the absorption of light that can be converted into electrical current in thin film solar cells is crucial for enhancing the overall efficiency and in reducing the cost. Therefore, light trapping strategies play a significant role in achieving this goal. The main objectives of light trapping techniques are to decrease incident light reflection, increase the light absorption, and modify the optical response of the device for use in different applications. Nanostructures utilize key sets of approaches to achieve these objectives, including gradual refractive index matching, and coupling incident light into guided modes and localized plasmon resonances, as well as surface plasmon polariton modes. In this review, we discuss some of the recent developments in the design and implementation of nanostructures for light trapping in solar cells. These include the development of solar cells containing photonic and plasmonic nanostructures. The distinct benefits and challenges of these schemes are also explained and discussed.

## 1. Introduction

The current world energy generation system is unsustainable, insufficient, cost-ineffective, and environmentally unfriendly. A number of alternative energy production from renewable sources such as solar, wind, hydroelectric, tidal, bioenergy, and geothermal have been extensively explored. The renewable energy sources are free and abundantly available and most important do not harm the environment. Solar energy is one of the promising alternatives to replacing fossil fuel among other energy sources because it has the potential to meet future energy demands at low cost with no detrimental effects to the environment. There are different technologies to harvest solar energy, and typical examples include solar electric (photovoltaic), solar thermal conversion, and solar fuel technologies [1]. Photovoltaic energy conversion, which converts light energy directly into electricity without any intermediate stage, has already demonstrated its success and widespread applications for solar energy utilization. The photovoltaic market has shown very significant yearly growth rates and the total global installed solar photovoltaic (PV) capacity had grown to over 500 GW by the end of 2018 [2]. A projected additional 500 GW of PV capacity is expected to be installed by 2023, driven by greater cost reduction and higher demand. Currently, more than 90% of the global photovoltaic solar cell market is dominated by crystalline Si-based solar cells. This is contrasted with less than 10% of other technologies based on thin films of cadmium telluride (CdTe), amorphous silicon (a-Si:H), microcrystalline silicon (µc-Si:H), and copper indium gallium selenide (CIGS) [3]. 

In order to meet the requirements of the global energy demand using photovoltaics, further conversion efficiency improvements and reductions in production costs are necessary. The use of advanced nanophotonic light trapping approaches can contribute to both objectives simultaneously. Various light trapping techniques have been implemented over the last few years to increase the light absorption within the semiconductor layer that is much smaller compared to the material’s intrinsic absorption length [4,5,6,7,8,9,10,11]. Enhancing the optical absorption also allows for decreasing the active absorber layer thickness, which in turn decreases the production costs through the use of significantly less materials. In addition, light trapping can enhance solar cell efficiency as thinner devices offer improved photogenerated charge carrier collection with less constraints on the diffusion lengths, potentially a higher open-circuit voltage, and improved stability through texturing material encapsulation [12,13,14]. In addition, incident light reaching the active absorber layer in solar cells can be interfered by dust particles in typical terrestrial environments and thus decreases the power conversion efficiency. Therefore, nanophotonic structures with a self-cleaning capability coating the solar cell are becoming necessary for sustainability and improved performance of the solar cells in typical terrestrial environments [15,16,17,18,19,20,21,22]. 

Minimizing optical losses such as reflections from front surfaces, preventing light from entering the solar cell active material, and poor absorption due to the transmission, particularly in thin film solar cells, have long been the main challenge in increasing the conversion efficiency. Light trapping structures are needed to maximize the optical path length of sunlight into solar cells through multiple passes and reduce reflections as they act as an antireflection coating in order to enhance the overall efficiency. Typically, a thicker active layer can improve the absorption of more sunlight. However, the optical thickness of the active absorber layer can be increased several times by the use of light trapping structures in a solar cell while its physical thickness remains unchanged. 

In general, light trapping techniques have been utilized in the development of high performance and low-cost solar cells by enhancing light absorption without requiring thicker active layers. The most widely used light trapping techniques in the industry are the upright or inverted pyramid structure [23,24] or random textures [25] with a typical characteristic feature size 3–10 µm utilized mainly for texturing crystalline silicon solar cells [26]. Such micron-scale features are not beneficial for thin film solar cells in which the active absorber layer is just a couple of microns or even several hundred nanometers in thicknesses. In addition, micron-scale features require deep etching and are known to introduce defects in the material [27]. 

Therefore, nanostructures are needed in order to apply light trapping in thin films and emerging low-cost solar cells. The use of nanoscale surface structures for improving light absorption of thin film solar cells is a promising method compared with the traditional micro-sized surface texturing for crystalline silicon solar cells [28,29]. This is because of the reduced etching depths required to form the nanoscale features and consequently decrease the level of damage to the substrates [30]. Furthermore, reflections are reduced over a wide range of wavelength in sub-wavelength nanophotonic structures. It has also been theoretically illustrated that nanophotonic structures can achieve optical path length enhancement beyond the Yablonovitch conventional limit [31]. The light trapping in thin film solar cells can be achieved using various nanostructures. The most widely recognized approaches for light trapping in thin film solar cells can be listed as periodic grating structures [32,33,34,35], photonic crystal structures [36,37,38,39], nanowires [40,41,42], random scattering surfaces [43,44], and plasmonic structures [45,46,47].

In this article, we review some of the recent developments in the design and implementation of nanostructures for light trapping in solar cells. This includes geometric engineering of the solar cell and the implementation of photonic and plasmonic nanostructures. The distinct advantages and challenges of these strategies are also discussed.

## 2. Photonic Nanostructures

**Figure 1:**
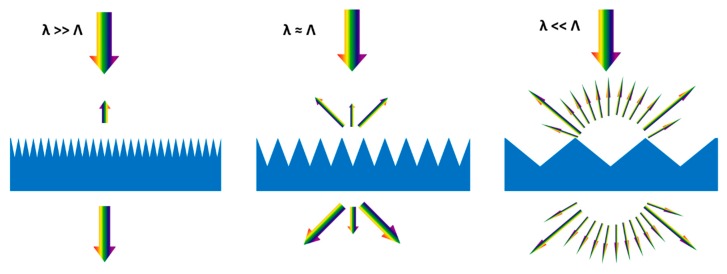
Schematic illustration of the optical effects induced for a specified wavelength by periodically textured surfaces of changing unique frequency. λ is wavelength and Λ is the structure period.

The periodic structures incorporated into a solar cell surface can contribute to both reducing reflection and enhancing the optical path length of light. However, certainly both impacts cannot be utilized simultaneously, depending on the place (front or back side of the cell surface), type, and size of the surface structure. Figure 1 illustrates the optical impacts of textured surfaces. These are typical cases of three light wavelengths λ incident on structures with period Λ, smaller, equal to, or larger than λ. 

A large number of diffraction orders can propagate through the textured structure for structure sizes larger than the wavelength (λ << Λ). The textured surface shape heavily affects the intensity spreading of these higher diffraction orders. This impact provides a guide to the geometric optical limit of refraction for a very high ratio of structure period to the wavelength that can be defined by Snell’s law. The optical path length within the active volume of solar cells can be enhanced owing to these effects linked to a change of propagation direction. Another effect is that various reflections can happen geometrically in these large textured surfaces. Thus, the overall reflection can be additionally decreased at the front surface. 

The interference effects that lead to specific diffraction orders influence the optical properties for wavelengths and structure sizes of similar dimensions (λ ≈ Λ). The response in terms of transmission and reflection properties of the textured surface depends strongly on wavelength due to such effects. Both reduction in reflection and significant enhancement in optical path length can be achieved by proper engineering of structure geometry. 

The effective medium theories can be used to explain the optical properties of periodically textured surfaces with structure sizes much smaller than the incident light wavelengths [48]. The theory suggests that the light does not pass through these structures and thus behave as an effective refractive index medium. These small size structures affect reflections and transmission but do not cause any light guidance impact as there is no change in light propagation direction. Thus, it is possible to achieve a very effective and broadband antireflection effect with these structures. 

**Figure 2:**
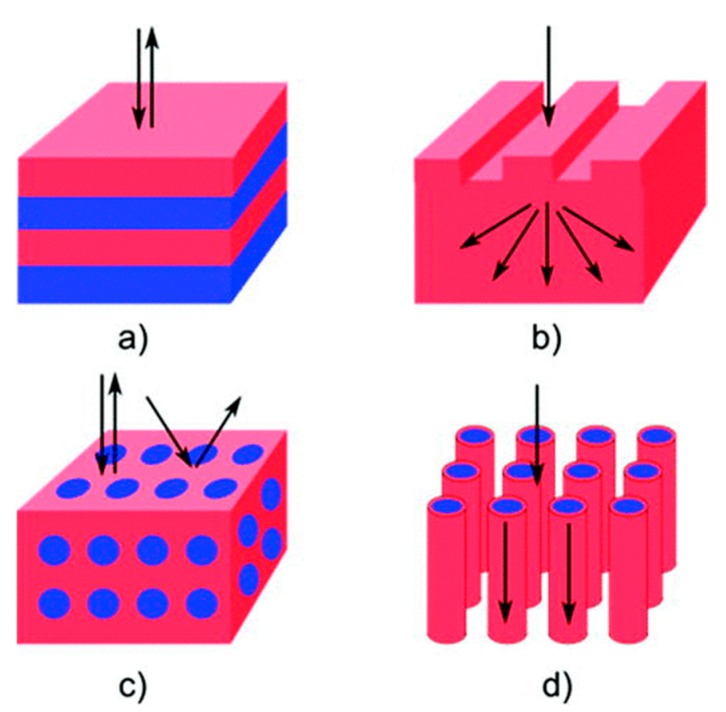
Schematic illustration of nanophotonic structures used for enhancing solar cell performance: (**a**) 1D (Bragg) stacks, (**b**) 2D gratings, (**c**) photonic crystal, and (**d**) nanowires. Reprinted (adapted) with permission from [49]. Copyright (2012) AIP Publishing.

One, two, or three dimensional (1D, 2D, or 3D) periodic nanostructures or gratings are promising for achieving light trapping in solar cells and hence enhancing their efficiencies. Figure 2 illustrates a schematic diagram of photonic nanostructures used in several configurations to improve solar cell performance. 

The optical path length of light in the active absorber layer can be doubled by using optimized 1D dielectric gratings or Bragg stacks as back reflectors. The reflection can be reduced from the illuminated surface of the solar cells or light can be trapped inside the active absorber layer using single or bi-periodic dielectric structures [49]. Two-dimensional sub-wavelength gratings are even more promising than one-dimensional gratings since the reflectivity does not depend on the polarization of the incident light [50]. In tandem solar cells, 3D periodic nanophotonic structures or photonic crystals can be employed as vastly efficient omnidirectional reflectors [39].

A variety of nanophotonic structures, such as nanocones [51,52,53,54], nanorods [55,56,57], nanopillars [58,59,60], nanowells [61,62,63], nanopyramids [64,65,66,67,68,69,70], and nanospheres [71,72], have been extensively studied for enhancing the performance of the solar cells. The photonic nanostructures themselves can be dielectric, metallic, or the absorber layer itself [71,73,74]. Likewise, nanostructures can be generated at the bottom and/or top surface of the active absorber layer or embedded within the active absorber region.

### 2.1. Nanostructures at the Front Surface 

**Figure 3:**
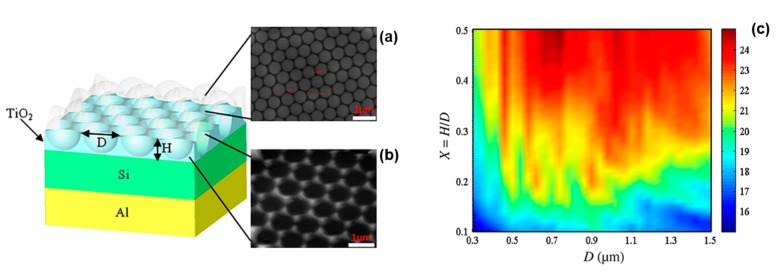
The periodic nanoisland structure on front surface of thin film silicon with an aluminum back-reflector. (**a**) SEM image of periodically arranged polystyrene spheres in a hexagonal lattice, (**b**) the remaining nanoislands after titanium dioxide (TiO_2_) deposition and nanosphere lift-off, and (**c**) the calculated short-circuit current density Jsc (mA/cm^2^) for a 2-μm-thick thin film crystalline Si as a function of structural parameters. Reprinted (adapted) with permission from [75]. Copyright (2011) John Wiley and Sons.

Nanostructures can be used at the front surface of the solar cell to offer an efficient pathway to couple the incoming light into the absorber layer and thus reduce reflection. For example, a light trapping element that consists of a periodic nanoisland structure formed on the front surface of thin film silicon fabricated by polystyrene colloidal lithography (Figure 3) was demonstrated [75]. In this design, the nanoisland shape not only exhibited gradual refractive index matching for antireflection but also enhanced the light trapping through diffraction of incident light in a periodic structure. Here, careful engineering of the dimensions of the nanoisland structures can assist in further improving the flow of light into the absorber layer. The solar cell with optimized parameters of a periodic nanoisland structure provides theoretically the largest short-circuit current density of 25 mA/cm^2^, which is a significant 76.9% increase compared with that of a bare thin c-silicon solar cell. Furthermore, the nanoisland structures contribute to the enhancement of the photocurrent densities at large angles of incidence, as compared to conventional antireflection coatings.

It has also been demonstrated that placement of a periodic array of resonant dielectric nanospheres on a top of an a-Si layer supporting whispering gallery modes significantly enhances the efficiency of a thin film a-Si solar cell [71]. Wavelength-scale resonant dielectric nanospheres can diffractively couple the incoming light from free space and also assist confined resonant modes. In addition, a periodic array of dielectric nanospheres can lead to light coupling between the spheres due to whispering gallery resonances within the spheres [76,77]. The optical path length of light inside the active material can be enhanced due to the light coupling into resonant modes when dielectric nanospheres are close to the absorber material and thus significantly improving light absorption. Another significant advantage of the nanosphere structure for solar cell application is its spherical shape that naturally admits light from large angles of incidence.

**Figure 4:**
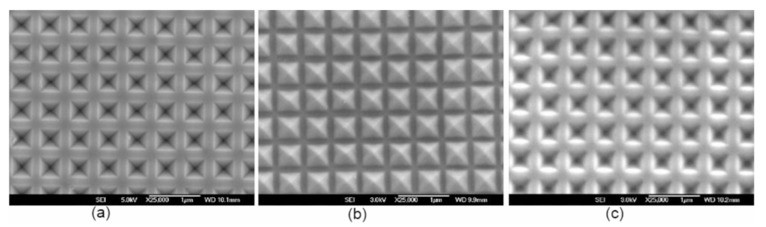
SEM image of (**a**) the periodic inverted nanopyramid structures on Si master mold, (**b**) the periodic upright nanopyramid structures replica after the first imprint, and (**c**) the periodic inverted nanopyramid structures fabricated on the surface of the solar cells after the second imprint. Reprinted (adapted) with permission from [35]. Copyright (2017) Elsevier.

We reported [35] that significant improvements in photocurrent and power conversion efficiency were achieved for monocrystalline silicon solar cells with periodic inverted nanopyramid structures due to the reduction of reflections and entrapment of more incident light inside the active material. The periodic inverted nanopyramid structures were fabricated by UV nanoimprint lithography using Si master mold, which was fabricated by laser interference lithography and subsequent pattern transfer by combined reactive ion etching and KOH wet etching (Figure 4). The solar cell with periodic inverted nanopyramid structures showed an improvement in power conversion efficiency by 11.73% compared to the planar solar cells.

It has been reported that high refractive index dielectric nanostructures supporting Mie resonances can be utilized to achieve very efficient light trapping in solar cells [78,79,80,81]. The excitation of Mie resonance leads to strong forward scattering of incident light into the higher-index absorber layer due to the high optical density of states. For example, Spinelli et al. [78] showed experimentally that an array of Si nano-cylinders (NCs) etched into a Si wafer supporting Mie resonances decreased the reflectivity of the Si down to 1.3% in the 450–900 nm wavelength range, and for angles of incidence up to 60. It was demonstrated theoretically that light trapping can be enhanced with silicon-on-insulator (SOI) wafers decorated with arrays of subwavelength light funnels (LFs) [82,83]. The mechanism behind the enhanced light absorption is the preferential forward scattering of light due to excitation of Mie modes in the arrays of light funnels.

Various other types of nanostructures on the front surface, such as nanopillars [60], nanowells [61], nanowires [84], and nanocones [53], have gained substantial attention due to their outstanding ability to reduce optical reflection from properly engineering the surface structure to produce graded refractive index structures. Various techniques can be used to fabricate nanostructures for light trapping such as nanosphere lithography (NSL) [85], colloidal lithography [75], electron beam lithography (EBL) [50], laser interference lithography (LIL) [35], and nanoimprint lithography (NIL) [86]. Nanostructure fabrication should be low-cost, and have high throughput, high fidelity, and be scalable for application in commercial photovoltaic technologies. NIL is one of the most cost efficient nanopatterning methods.

### 2.2. Nanostructures at the Back Surface

**Figure 5:**
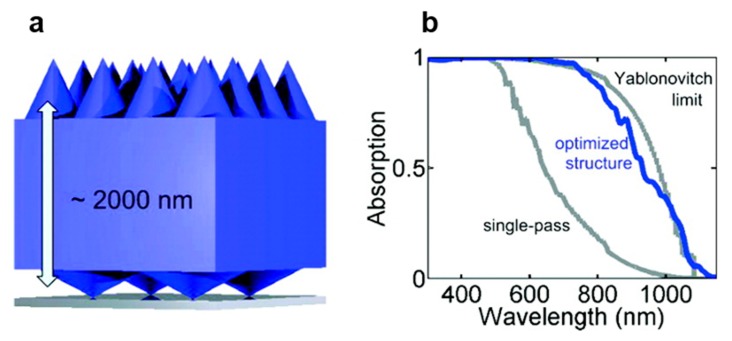
(**a**) Schematic of a both-sided nanocone grating design of an ultrathin film Si solar cell, where the front and back surfaces of the cell were separately optimized for antireflection and light trapping, respectively, and (**b**) the spectral absorption of the optimized structure as a function of wavelength. Reprinted (adapted) with permission from [91]. Copyright (2012) American Chemical Society.

Nanostructures can be utilized at the back surface of an absorber layer as high performance reflectors of light. A highly promising approach is to use a periodic array of nanostructured back reflectors (photonic crystal) to couple incident light into guided modes, propagating in the absorber plane [32,87,88]. Careful tuning of the shape and periodicities of the nanostructures offers a new degree of control across the polarization and angular distributions of the scattered light. This strategy is capable of significantly improving the optical path length within the absorber layer. A broad range of nanostructure shapes, dimensions, and periodicities have been investigated to optimize light trapping in thin film solar cells [89,90,91]. For example, systematic rigorous coupled wave analysis (RCWA) was performed to investigate the possible benefits of nanostructured double-side nanocone grating of an ultrathin c-Si solar cell, as shown in Figure 5 [91]. 

From this study, it was found that significant absorption enhancement can be achieved if high-aspect-ratio, dense (periodicity ~500 nm) nanocone grating is utilized in the front surface as an antireflection, and low-aspect-ratio lower-density nanocone grating are utilized in the back surface to allow the coupling to guided resonances. Here, the optimal periodicity for light tapping was found to be close to the targeted wavelength for a 2 µm thin Si cell. The optimum periodicity of the nanocones on the back surface was determined to be 1000 nm as light trapping is more important for the 800 to 1100 nm wavelength range, near the bandgap of Si.

### 2.3. Nanostructures at the Absorber Layer

When the nanostructures are introduced into the semiconductor layer, they can provide an efficient way to enhance both the optical and electronic properties of the solar cells. For example, solar cells consisting of arrays of Si nanowires with radial p-n junctions that offer broadband optical absorption properties and efficient charge carrier collection along the length of the wire [6,92,93,94]. The direction of carrier and photon transport are orthogonal in a radical p-n junction, which allows for effective photogenerated carrier collection from low-quality materials with short minority-carrier diffusion length, while enabling for high optical absorption and external quantum yields for collection of the charge carrier. Both the light absorption and charge carrier collection of the nanowire array devices are strongly dependent on the spacing, orientation, and size of the wire [95,96]. Detailed joint optimization of both these properties is required to maximize the efficiency of the solar cell for nanowire array devices. 

**Figure 6:**
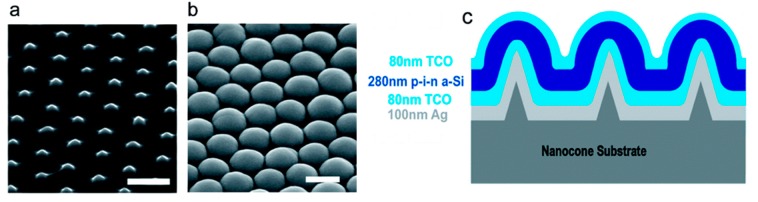
SEM images at 45° on (**a**) nanocone patterned quartz substrate and (**b**) a-Si:H nanodome solar cells after deposition of all layers on nanocones (scale bar 500 nm). (**c**) Schematic illustration of the cross-sectional view of a-Si:H nanodome solar cells. Reprinted (adapted) with permission from [5]. Copyright (2009) American Chemical Society.

It has been demonstrated that a nanostructured solar cell can be fabricated from the bottom patterned substrate, through the active absorber layer to the top contact, where all the layers have periodic nanostructures [97]. For example, nanodome a-Si:H solar cells were built by depositing the absorbing layer on nanocone patterned substrate as shown in Figure 6 [5]. The 280 nm-thick a-Si:H layer was conformally deposited on top of the nanocone patterned substrate, resulting in the active semiconductor layer with nanodomes. In this device, the tapered shape of nanodome structures not only offered better effective refractive index matching with air for antireflection but also coupled the incident light into strong guided modes in the a-Si:H layer, enhancing the optical path length for absorption. The antireflection effects play a significant role for short wavelength around 400–500 nm where a-Si:H is highly absorptive, and all the incident light can be absorbed in a single path. The incident light can be efficiently coupled into planar guided modes through the tapered shape of the nanodome for the long wavelength region (600–700 nm) where a-Si:H is less absorptive, and all the incident light cannot be absorbed in a single path. These nanodome solar cells combine antireflection and light trapping effects to both effectively decrease reflection and improve the absorption over a broad spectral range. It was also shown that the absorption of photons can be significantly increased by nanodome structures even for lower bandgaps of a-Si. The absorption with wavelengths of 400–800 nm (94%) significantly increased for nanodome a-Si:H solar cells compared to the absorption of planar solar cells (65%). The enhancement in the light absorption contributed to a final cell efficiency of 5.9%, which was 25% higher than a planar reference cell. 

The impacts of photonic crystals can also play a role when the periodic nanostructure size is comparable to or even smaller than the wavelength scale [39,72,98]. It was shown that the limit for the enhancement of light trapping is associated with the local photonic density of optical states [87]. The light trapping can be significantly improved for the absorber layer with photonic crystal where the local density of optical states is high [99,100,101]. This shows the potential of beyond the Lambertian light-trapping limit. Various types of photonic crystals consisting of periodically arranging strips [102], nanodomes [5], nanopillars [103], nanoholes [62,104], and nanowells [61] have been investigated for solar cells application.

## 3. Plasmonic Nanostructures

**Figure 7:**
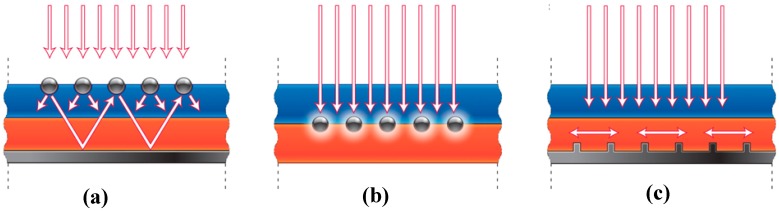
Schematic of three different plasmonic light-trapping geometries for thin film solar cells. (**a**) Metal nanoparticles placed on top of a solar cell, (**b**) metal nanoparticles embedded in the semiconductor, and (**c**) nanostructured metal films placed on the back surface of a solar cell. Reprinted (adapted) with permission from [110]. Copyright (2010) Springer Nature.

Plasmonics is based on the interaction between the electromagnetic radiation and conductive electrons in metal [105]. Surface plasmons can be either localized surface plasmons excited in metal nanoparticles [106,107] or propagating surface plasmon polaritons (SPPs) at a metal/semiconductor interface [108,109]. Plasmonic structures can be integrated into thin film solar cells in at least three different configurations for light trapping structures that can significantly reduce the photovoltaic absorber layer physical thickness while maintaining their optical thickness constant, as shown in Figure 7. 

In the first scheme, metallic nanoparticles can be used as subwavelength scattering elements to couple incident sunlight into an absorbing semiconductor layer. Properly engineered metallic nanoparticles can produce localized surface plasmons, which strongly scatter light into the guided modes of the substrate. If these particles are on top of the solar cells, as shown in Figure 7a, the optical path length of light can be increased inside the active absorber layer due to the scattered light. In the second scheme, metal nanoparticles are embedded as subwavelength optical antennas within the semiconductor material in which the plasmonic near-field is coupled to the semiconductor material, increasing absorption in surrounding regions of semiconductor material (Figure 7b). In the third scheme, nanostructured metal films placed on the back surface of the solar cell can couple propagating sunlight into surface plasmon polariton (SPP) modes (see Figure 7c). These modes propagate along with the metal and semiconductor interface, confining the light along the boundary. 

### 3.1. Light Scattering Effect

The scattering cross sections can be improved with metal nanoparticles at wavelengths close to the plasmon resonance as a result of a collective oscillation of the conduction electrons in the metal. The light scattering is nearly symmetrical in both backward and forward directions as a tiny nanoparticle is surrounded in a homogeneous medium [111]. This circumstance varies when the metal nanoparticle is positioned near the interface between the two dielectric materials; in this situation light scatters favorably into the dielectric material with higher permittivity [112]. The optical path length of the light can be effectively improved due to the scattered light acquiring an angular distribution in the semiconductor material. Furthermore, with the metallic reflector as a back contact of the solar cell, the light that is weakly absorbed in a single pass can be reflected towards the surface and be partly reradiated into the semiconductor layer by the nanoparticles. In addition, light scattered at an angle exceeding the critical angle of reflection remains trapped within the solar cell. Thus, the optical path length can be efficiently increased as the incident light travels through the active layer multiple times, which improves the probability of scattered light to be absorbed and generates more charge carriers.

Stuart and Hall first demonstrated that light coupling can be improved in a semiconductor thin film with plasmonic nanoparticles due to resonant scattering [113,114]. They reported that a 165 nm silicon-on-insulator photodetector with silver metal-island films deposited onto the device showed an enhancement in photocurrent nearly by a factor of 20 at a wavelength of 800 nm. The far-field scattering approaches from subwavelength metallic nanoparticles were extensively investigated in thin film and emerging solar cells [115,116,117,118,119]. For instance, amorphous silicon p-i-n solar cells with Au nanoparticles deposited onto the top surface of amorphous silicon film showed improvements in photocurrent and power conversion efficiency by 8.1% and 8.3%, respectively [120]. Thin GaAs solar cells deposited with size-controlled Ag nanoparticles on top of the device surface showed an enhancement in light absorption for wavelengths longer than 600 nm and improvement in short circuit current density by 8% [117]. Pillai et al. [107] found that 1.25 µm thin silicon on insulator solar cells with silver nanoparticles exhibited an enhancement in light absorption over a broad wavelength range and enhancement at 1050 nm wavelength by a factor of 16 due to the effect of scattering.

However, plasmonic nanostructures suffer from parasitic absorption that can severely limit the overall photocurrent improvement; thus, the performance of the solar cell with plasmonic nanostructures is limited [121,122,123]. The optical properties of the plasmonic nanoparticles rely strongly on their material, shape, size, and refractive index of the medium, and distance from the semiconductor material [124]. Here, the use of plasmonic nanostructures for solar cells should be properly engineered to improve the scattering and reduce the parasitic losses over a broad wavelength range significant for light trapping. All of the nanoparticle arrays provide enhanced scattering effects at long wavelengths, whereas parasitic absorption in Au and Ag nanoparticle arrays is relatively high at short wavelengths due to the Fano effect, limiting the device performance. However, parasitic losses can be mitigated through Al nanoparticles due to their surface plasmon resonance lying in the ultraviolet range and scattering effects dominating a broadband spectral range [125,126,127]. Al nanoparticles have been applied to various types of solar cells for efficient light trapping [128,129,130,131]. It has been theoretically observed that Si wafers, by incorporating tailored Al nanoparticles, allow broadband light trapping, resulting in 28.7% increase in photon absorption, which is much higher than that produced by Ag or Au [128].

**Figure 8:**
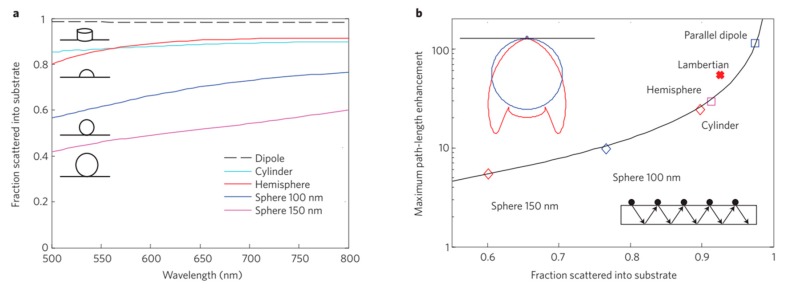
(**a**) Fraction of light scattered into the substrate, divided by total scattered power, for different sizes and shapes of Ag particles on Si. (**b**) Maximum path-length improvement for the same geometries as in (**a**) at a wavelength of 800 nm. Reprinted (adapted) with permission from [132]. Copyright (2008) AIP Publishing.

Catchpole et al. [132] demonstrated that enhanced near-field coupling resulted in higher optical path length improvements for cylindrical and hemispherical particles compared to the spherical particles (Figure 8). It was found that changing the distance of the particles from the substrate could manipulate the scattering cross section of the nanoparticles. They also reported that silver nanoparticles provided much higher path length improvements than gold particles.

### 3.2. Near-Field Effect 

The strong local field enhancement around the metal nanoparticles from localized plasmon resonances can be efficiently utilized in thin film solar cells. Small metallic nanoparticles are embedded in the active material, which acts as optical antennas for incident light that stores the incident energy in the localized surface plasmon resonance. The excitation of surface plasmons can be absorbed in the surrounding active material due to the plasmonic near-field coupling and thus effectively enhances the light absorption in the solar cell. This plasmonic near-field effect can be strongly enhanced with small nanoparticles (5–20 nm diameter) for which far-field scattering is low [133,134]. This near-field mechanism works particularly well for materials with short carrier diffusion lengths, and electron–hole pairs must, therefore, be generated in the vicinity of the collection junction area. The light absorption due to the plasmonic near-field coupling is highly dependent on the size and shape of the metal nanoparticles, the spacing between neighboring nanoparticles, the coating thickness, and the dielectric medium of the embedding layer [135,136,137,138]. 

The enhanced photocurrents due to the plasmonic near-field coupling have been widely investigated for both inorganic and organic solar cells [133,139,140,141]. The absorption enhancement over a broad spectral range and improved device efficiency have been demonstrated for tandem ultrathin film organic solar cells consisting of an array of approximately 5 nm diameter silver nanoparticles [140]. Organic solar cells, by integrating electrodeposited Ag nanoparticles, showed enhanced efficiencies from 3.05% to 3.69% due to improved absorption of the active material [142]. Organic bulk heterojunction solar cells containing plasmon active silver nanoparticles showed an enhancement in device efficiency by a factor of 1.7 [143]. An increase in efficiency was reported for dye-sensitized solar cells by embedding small metal nanoparticles [144,145,146]. Significant improvements in both the photocurrent (14.1%) and fill factor (12.3%) were achieved for a-Si solar cells with ultra-small metallic nanoparticles owing to the strong plasmonic near-field concentration and the reduced contact resistance, respectively [147]. 

### 3.3. Surface Plasmon Polariton Modes

Another approach investigated plasmonic light trapping design, where light was converted into SPPs, which are electromagnetic waves that propagate along with the metal back contact/semiconductor layer interface for a relatively long distance [148]. Close to the plasmon resonance wavelength, the evanescent electromagnetic SPP waves are confined to the subwavelength scale near the interface. The incident light can be effectively trapped and guided into the semiconductor absorber layer owing to SPPs excited at the interface between the metal and semiconductor layer. In this design, incident light is efficiently turned by 90° and absorbed in the semiconductor absorber layer that enhances the optical path length by several orders of magnitude with respect to the thickness of the semiconductor absorber layer. The absorption of SPPs in the semiconductor absorber materials must be higher than that of the metal back contact, which is beneficial for efficient light absorption. However, these enhanced plasmon trapping effects are limited due to the parasitic absorption in metal. By proper engineering of metal geometry and choice of materials, the best tradeoff between decreased plasmon losses and pronounced field confinement can be found.

Several reports on the integration of SPP architectures have been already realized and investigated for ultrathin solar cells, including organic solar cells [149,150,151,152,153]. For example, Ferry et al. [154] designed the periodic nanostructured plasmonic back contacts by nanoimprint lithography for an a-Si:H solar cell, which showed a 26% enhancement in short circuit current density. The photocurrent enhancement occurred predominantly in the spectral range from 600 to 800 nm with an active layer thickness of less than 200 nm where SPPs modes were supported at the metal interface. Lee et al. [155] demonstrated a tunable resonance into the absorption spectra through the hybridization between the localized surface plasmons and the SPP modes in nanovoids in an organic (P3HT:PCBM) plasmonic solar cell. A 33% relative absorption enhancement was first achieved by standard grating structures in the short wavelength region. They then introduced the nanovoids into an optimized rectangular grating structure, which enhanced the absorption resonance in the long wavelength region, leading to a 41% relative absorption enhancement.

As discussed above, the light absorption is very weak for long wavelengths with the photonic nanostructures at the front side of the solar cells for thin film solar cells. On the other hand, plasmonic nanostructures at the back surface of the solar cells provide a stronger light absorption improvement for the long wavelengths with negligible effects on the short wavelengths compared with plasmonic nanostructures at the front surface of the solar cells. Thus, a broadband light absorption enhancement for thin film solar cells can be potentially achieved by incorporating both photonic nanostructures at the top side of the cells and plasmonic nanostructures at the back side of the cells. Zhang et al. [156] demonstrated a hybrid structure incorporating both the biomimetic silicon moth-eye structure at the top surface of the cells and Ag nanoparticles at the back side of the cells to achieve a broadband light absorption in 2 μm thick crystalline silicon solar cells reaching the Yablonovitch limit. It has been found that the solar cells with both silicon moth-eye and Ag nanoparticles achieved 69% light absorption enhancement over a broad spectral range compared to conventional light trapping structures. This is substantially higher than individual light trapping structures of silicon moth-eye (58%) and Ag nanoparticles (41%).

## 4. Summary

Advanced light trapping techniques are important for the development of thin film solar cells to obtain higher efficiency and lower cost. Nanostructures with unique scales and geometries are intended to play a significant role for light trapping in the subwavelength region. In this article, we reviewed the recent progress in the design and implementation of nanostructures for light trapping in solar cells. Many device architectures can be used to integrate photonic and/or plasmonic nanostructures in solar cells. These nanostructures provide enhanced antireflection, increased light absorption, and the ability to tailor the optical properties of solar cells to different applications in unprecedented ways. The light trapping photonic and plasmonic nanostructures have shown enhanced efficiency in many types of solar cells, but careful engineering and improved fabrication techniques can extract the full potential from these light trapping approaches with better electrical properties that may target broadband coverage of the solar spectrum.
